# Ovarian serous carcinoma in which mediastinal recurrence of the cancer was resected 16 years after surgery: A case report

**DOI:** 10.1002/rcr2.988

**Published:** 2022-06-09

**Authors:** Hiroyuki Miura, Jun Miura, Shinichi Goto, Tomoko Yamamoto

**Affiliations:** ^1^ Department of Thoracic Surgery Akiru Municipal Medical Centre Tokyo Japan; ^2^ Department of Surgery Kyorin University School of Medicine Tokyo Japan; ^3^ Department of Respirology Akiru Municipal Medical Centre Tokyo Japan; ^4^ Department of Pathology Tokyo Women's Medical University Tokyo Japan

**Keywords:** late recurrence, mediastinal recurrence, ovarian cancer, serous carcinoma

## Abstract

We report a rare case of ovarian carcinoma in which a mediastinal recurrence was resected 16 years after the initial operation. A 72‐year‐old woman underwent hysterectomy with adnexectomy for stage IIIC ovarian serous carcinoma after neoadjuvant chemotherapy. Six courses of adjuvant chemotherapy were administered. Three years after surgery, left supraclavicular lymph node metastasis occurred, and radiotherapy and two courses of chemotherapy were administered. Six years before presentation, a metastasis at the right cardiophrenic lymph node was resected, and six courses of chemotherapy were administered. During follow‐up, a retrosternal tumour was found. The metastatic lesion in contact with the diaphragm was thought to result from pleuroperitoneal communication, and it increased in size. Although high‐grade serous carcinoma is aggressive, its sensitivity to chemotherapy may suppress early recurrence, contributing to good outcomes, but with late recurrence. Multidisciplinary therapy including surgery is required for improved long‐term prognosis for mediastinal metastasis of ovarian serous carcinoma.

## Graphical abstract

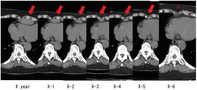

## INTRODUCTION

Recurrence of ovarian cancer often occurs in the abdominal cavity, but there are only a few reports of its recurrence in the thoracic cavity and mediastinum. Here, we report a case of ovarian serous carcinoma in which a mediastinal recurrence was resected 16 years after the initial operation.

## CASE REPORT

A 72‐year‐old Japanese woman presented with an abnormal shadow on chest computed tomography (CT). Sixteen years previously, peritoneal dissemination was observed when laparotomy was performed based on a diagnosis of ovarian cancer. After four courses of chemotherapy consisting of carboplatin and paclitaxel, hysterectomy with adnexectomy was performed. The pathological finding was stage IIIC serous cystadenocarcinoma. Six courses of adjuvant chemotherapy composed of the regimen mentioned above were administered. Three years after surgery, left supraclavicular lymph node metastasis occurred, and the patient was irradiated (70 Gy/35 fractions) after two courses of chemotherapy composed of carboplatin and paclitaxel. Six years before presentation, a metastatic lesion at the right cardiophrenic extrapulmonary area suspicious of lymph node metastasis was resected under the complete video‐assisted thoracic surgery, and six courses of chemotherapy also composed of carboplatin and paclitaxel were administered at another hospital.

**FIGURE 1:**
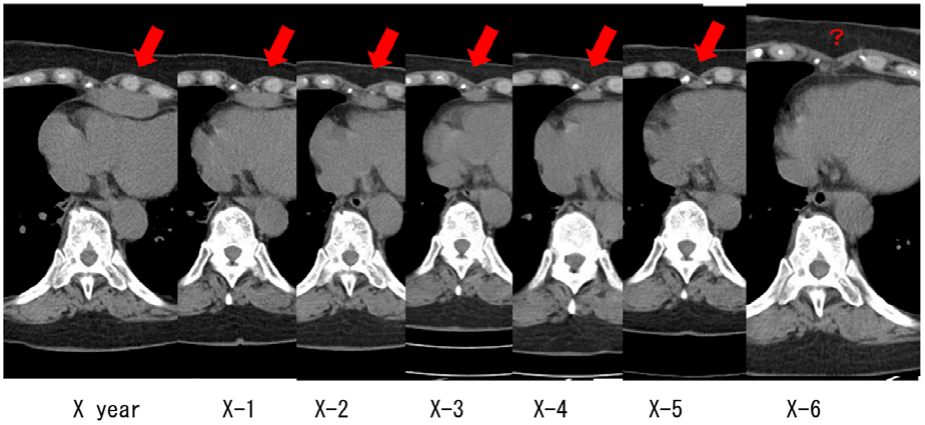
Chest computed tomography image showing a mediastinal tumour over 6 years

**FIGURE 2:**
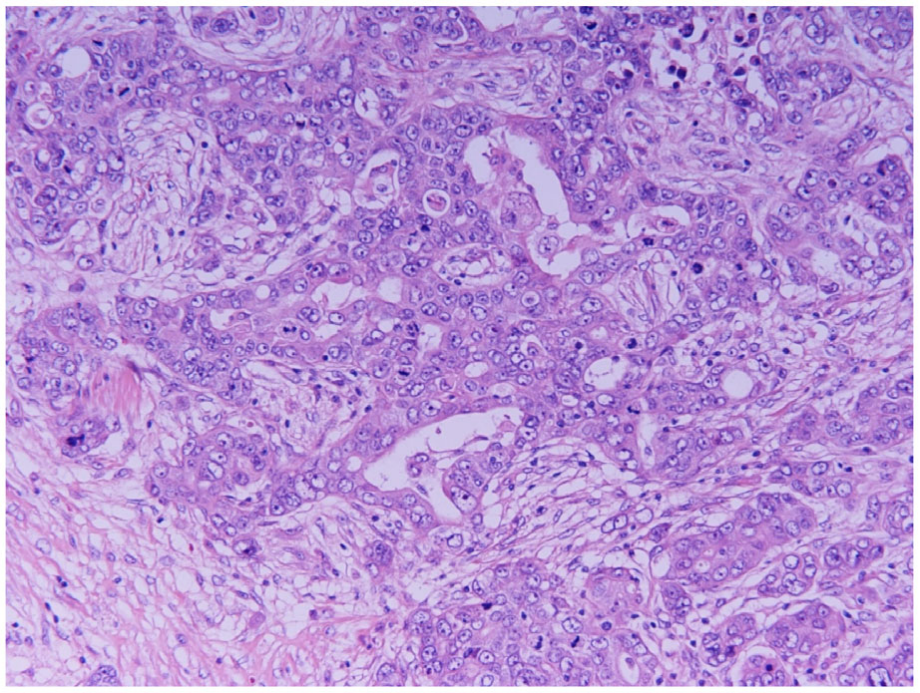
Histological findings of mediastinal tumour resembled those of primary ovarian serous carcinoma

During follow‐up, a tumour that kept increasing in size was found in the inferior retrosternum of the anterior mediastinum, and the patient was referred to our department. Chest CT revealed a tumour sized 52 × 21 mm that was not detected 6 years previously (Figure 1). Thymoma or ovarian cancer metastasis was suspected. The tumour was removed via a median sternotomy; it was strongly adhered to the diaphragm, which was resected along with the tumour. The tumour was an adenocarcinoma, with a similar histology to that of the ovarian cancer resected 16 years ago (Figure 2). Immunostaining revealed that the mediastinal metastasis of ovarian cancer that was p53‐positive, WT1‐positive, partially ER‐positive and napsin A‐negative. The stainability was same as that of the ovarian cancer. No obvious infiltration was observed in the diaphragm. Six courses of adjuvant chemotherapy with carboplatin and paclitaxel as well as radiotherapy were administered, and no metastasis or recurrence was observed 10 months after the operation (Table 1).

**TABLE 1:** Timeline of treatment

Date	Clinical findings	Treatment
X‐16	Ovarian cancer	Laparotomy; peritoneal dissemination Four courses of chemotherapy (CBDCA + PAC) Hysterectomy + adnexectomy
X‐13	Left supraclavicular LN metastasis	Two courses of chemotherapy (CBDCA + PAC) Radiotherapy 70 Gy/35 fractions
X‐6	Metastasis at the right Cardiophrenic area	Resection Six courses of chemotherapy (CBDCA + PAC)
X	Mediastinal metastasis	Resection

Abbreviations: CBDCA, carboplatin; LN, lymph node; PAC, paclitaxel.

## DISCUSSION

Serous carcinoma is the most common histological type of ovarian cancer, accounting for 40% of all cases of ovarian cancer. A recent study showed that it originates from the fallopian tube epithelium and secondarily involves the ovary.^1^ Serous carcinoma is classified into low‐grade and high‐grade types. The former exhibits slow progression with borderline malignancy as a precursor lesion, and the prognosis is good. The latter has no precursor lesions and exhibits aggressive progression, often leading to peritoneal dissemination, and it has a poor prognosis.^1^ Our case had a pathological diagnosis of high‐grade serous carcinoma.

To our knowledge, there are only seven reports of mediastinal metastasis from ovarian cancer other than mediastinal lymph nodes metastasis, including our case, and all seven were cases of serous carcinoma (Table 2). The reports clearly stated that mediastinal lymph node metastasis were excluded. In all cases but one, thymic metastasis was in contact with the diaphragm. One case involved a cystic mass abutting the pericardium in the middle mediastinum.^5^ A tumour reported by Lu and Goldblatt^3^ was speculated to invade to the diaphragm according to their operative findings. Of the seven cases, tumours were present in the cardiophrenic area in three cases. In two cases including our case, a tumour was found in the anterior mediastinum at the inferior retrosternum. In our case, a tumour located at the right cardiophrenic lesion suspicious of lymph node metastasis was found 6 years previously and was resected at another hospital. Lymph nodes were originally found at the site of the cardiophrenic lesion, and the tumour that metastasized to this lymph node may have increased in size and replaced the lymph nodes. Lopes et al. performed cardiophrenic lymph node resection in 29 of 456 patients with stage IIIC–IV epithelial ovarian carcinoma and found lymph node involvement in 24 patients.^8^ Twenty‐two of the cases were high‐grade serous carcinoma. In addition to lymph node metastasis, the metastatic lesion in contact with the diaphragm was thought to be due to pleuroperitoneal communication and the migrated tumour cells attached to the diaphragm.

**TABLE 2:** Mediastinal metastasis from ovarian cancer

First author	Histological type	Time to recurrence (years)	Part of mediastinum with recurrence	Recurrence site	Number of metastases	Treatment
Montero^2^	Low grade Serous	0	Anterior	Inferior Retrosternum	Oligo	Resection
Lu^3^	Papillary Serous	>20	Anterior	Cardiophrenic ~Thymus	Multiple	Resection
Chase^4^	Psammoma Serous	0	Anterior	Cardiophrenic ~Superior mediastinum	Multiple	Hormonal Cyberknife
Scarci^5^	High grade Serous	0	Middle		Oligo	Resection Chemotherapy
Inoue^6^	Papillary Serous	29	Anterior	Cardiophrenic	Oligo	Resection Chemotherapy
Omura^7^	Low grade Serous	33	Anterior	Thymus	Oligo	Resection
Present	High grade Serous	16	Anterior	Inferior Retrosternum	Oligo	Resection Chemotherapy

Inoue et al. reported a case of ovarian serous carcinoma that metastasized to the anterior mediastinum 29 years after the initial treatment.^5^ In addition, they referred to the literature about ovarian cancer that recurred more than 20 years after surgery, and all of them involved serous carcinoma.

Although high‐grade serous carcinoma is aggressive, it is responsive to chemotherapy. Conversely, chemotherapy was not associated with improved survival for low‐grade serous carcinoma.^9^ Serous carcinoma is common in epithelial ovarian cancer, and its sensitivity to chemotherapy may suppress early recurrence, contributing to good outcomes but with late recurrence. Multidisciplinary therapy including surgery is required for improved long‐term prognosis in mediastinal metastasis of ovarian serous carcinoma.

## AUTHOR CONTRIBUTION

Dr Hiroyuki Miura and Dr Shinichi Goto helped in the conception and design of the work and the acquisition and analysis or interpretation of data for the work. Dr Jun Miura drafted the work and revised it critically for important intellectual content. Dr Tomoko Yamamoto diagnosed this cancer pathologically. All authors contributed to the final version of this manuscript and approved it to be published.

## CONFLICT OF INTEREST

None declared.

## ETHICS STATEMENT

Appropriate written informed consent was obtained for the publication of this case report and accompanying images.

## Data Availability

The data that support the findings of this study are available on request from the corresponding author. The data are not publicly available due to privacy or ethical restrictions.
